# Pediatric infection with the Omicron variant increases the risks of febrile seizures among COVID-19 infected children

**DOI:** 10.3389/fped.2023.1226403

**Published:** 2023-08-17

**Authors:** Zakaria Ahmed Mohamed, Chunjiao Tang, Erick Thokerunga, Youping Deng, Jingyi Fan

**Affiliations:** ^1^ Department of Pediatrics, Zhongnan Hospital of Wuhan University, Wuhan, China; ^2^ Department of Clinical Laboratory Medicine, Center for Gene Diagnosis, Zhongnan Hospital of Wuhan University, Wuhan, China

**Keywords:** febrile seizure, SARS-CoV-2, children, Omicron variant, COVID policy

## Abstract

**Background:**

The Omicron variant of the severe acute respiratory syndrome coronavirus 2 (SARS-CoV-2) is less likely to cause severe disease in children than the other variants but has become an increasing cause of febrile seizures (FS) among children. In this case-control study, we aimed to examine the risk factors associated with FS in children infected with the COVID-19 Omicron variant and related treatment modalities.

**Methods:**

This retrospective case-control study includes 113 subjects infected with the COVID-19 Omicron variant, grouped into 45 cases (those with FS) and 68 controls (those without FS). Data on clinical features, laboratory parameters, and treatment modalities were collected and analyzed.

**Results:**

Approximately 5.74% of COVID-19 infected children developed COVID-19-associated FS. Children with COVID-19 and high body temperatures [RR 1.474; (95% CI: 1.196–1.818), *p* < 0.001], previous history of FS [RR 1.421; (95% CI: 1.088–1.855), *p* = 0.010], high procalcitonin levels [RR 1.140; (95% CI: 1.043–1.246), *p* = 0.048] and high neutrophil counts [RR 1.015; (95% CI: 1.000–1.029), *p* = 0.048] were more likely to experience FS than the controls. In contrast, children with COVID-19 and low eosinophil counts, low hemoglobin levels, and cough had a lower risk of developing FS [RR 0.494; (95% CI: 0.311–0.783), *p* = 0.003], [RR 0.979; (95% CI: 0.959–0.999), *p* = 0.044]; and [RR 0.473 (95% CI 0.252–0.890), *p* = 0.020]; respectively. Children with FS received more anti-flu medications than those without.

**Conclusion:**

A significant increase in FS was observed in children with Omicron SARS-CoV-2 infection. A higher body temperature, a history of FS, a higher procalcitonin level, and a high neutrophil count were all associated with an increased risk of FS in children with COVID-19. The risk of developing FS was lower in children with COVID-19 and low eosinophil counts and hemoglobin levels than in those without.

## Introduction

On December 7, 2022, China abruptly amended its COVID-19 containment policy, ending the almost three-year-long zero Covid policy. This sudden shift in approach led to a surge in COVID-19 outbreaks countrywide, with the mutated Omicron variant as the dominant variant (1). Unlike its predecessor, the delta variant, Omicron was particularly associated with significantly increased pediatric admissions in China (2–4) and abroad (5–9). It further impacted other pediatric illnesses; for instance, several US investigators noticed a sharp rise in the incidence of Croup, a clinically diagnosed pediatric disease caused by virus-induced subglottic airway inflammation or laryngotracheobronchitis (10–13). Recent publications from Korea (14), Japan (15), and the USA (16) have all demonstrated an association between the Severe acute respiratory syndrome coronavirus 2 (SARS-CoV-2) Omicron variant and increased incidence of febrile seizures among children compared to the pre-Omicron stage. While emerging evidence clarifies this association, the clinical characteristics of these children and other associated factors are still largely undescribed.

Febrile seizure (FS) is a common neurological disorder affecting 2%–4% of children <5 years old who do not have any intracranial infections (17). Although its exact etiology is still unclear, it's strongly associated with the elevation of body temperature, certain vaccinations, previous history and family history of FS, maternal alcohol exposure, iron deficiency, and viral infections (18–20). Among the published viral causes of FS, adenoviruses, human herpesvirus-6, influenza, and rhinoviruses are the standout culprits (21, 22); for instance, evidence suggests that 15%–20% of children hospitalized with influenza A virus develop FS (23). In Wuhan City, the Omicron strains BA.1, BA.2, BA.5, and BF.7 were the dominant strains that significantly drove up pediatric admissions at our hospital in February 2023.

Given the reported association between the Omicron variant and increased incidence of FS, coupled with the paucity of studies on the risk factors associated with febrile seizures among children infected with the SARS-CoV-2 Omicron variant, we sought to evaluate SARS-CoV-2-Omicron infected children to ascertain their risks for developing febrile seizures and related treatment modalities.

## Materials and methods

### Study design and population

This study was a retrospective case-control design conducted at the pediatrics department of Zhongnan Hospital of Wuhan University, Wuhan, China, using medical records of pediatric patients below 14 years of age who were admitted due to COVID-19 in December 2022. The cases had febrile seizures, while the controls were febrile children without seizures. Febrile seizures were diagnosed based on the International Classification of Disease (ICD)-10 code of R56.0 (febrile seizure), while COVID-19 was diagnosed using polymerase chain reaction (PCR) or antigen test. Cases were children aged 0 months to 12 years presenting with febrile seizures as per the American Academy of Pediatrics (AAP) guidelines (24), while controls were children aged 0 months to 13 years presenting with fever but no seizures. Children with afebrile seizures, chronic neurodevelopmental problems, metabolic abnormalities, central nervous system infections, and diagnosed cases of other hematologic issues, those on an iron supplement, chronic systemic diseases, and very sick children were excluded from the study. Consecutive cases and concurrent controls were selected from the same setting in a ratio of 1:1. We defined a simple febrile seizure as a generalized tonic-clonic seizure lasting less than 15 min, occurring only once in 24 h in a child between 6 months and 5 years of age with a fever of 38°C. A seizure lasting more than 15 min in a child under 6 months or more than 5 years of age and occurring more than once in 24 h is a complex febrile seizure. Febrile status epilepticus is any prolonged or recurrent seizure lasting more than 30 min without returning to full consciousness.

### Data collection

Data collected on both cases and controls were: Clinical history, physical examination, relevant laboratory investigations, and treatment. Clinical characteristics include; age (in months), sex, underlying disease, previous febrile seizure history, and family history. Seizure features included; underlying disease, previous febrile seizure history, family history, seizure duration (in minutes), total seizure number in 24 h, and peak body temperature. Treatment provided included; anticonvulsant therapy, antipyretics, and other medications used.

### Data analysis

The Statistical Package for Social Sciences, SPSS (Version 27, IBM, Inc.), was used for the data analysis. Statistical analysis included descriptive statistics and a multivariate Poisson regression model to determine the adjusted risk ratio and independent risk factors for febrile seizures in children.

## Results

### Database cohort overview

**Figure 1:**
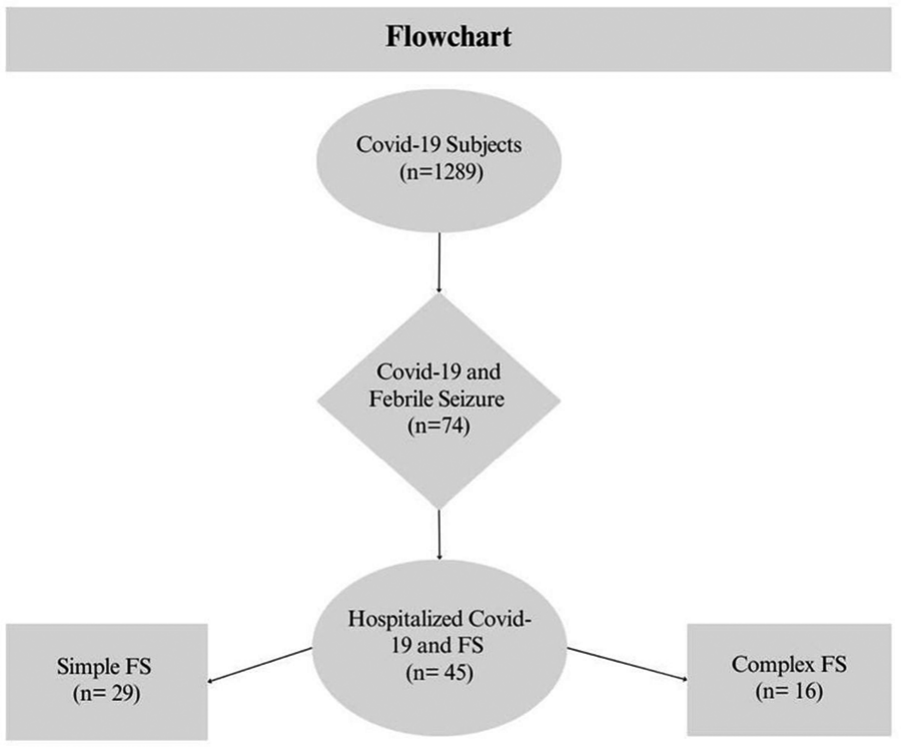
Database cohort overview flowchart.

In December 2022, 1,289 pediatric patients were diagnosed with COVID-19 in our outpatient department. Among these patients, 74 (5.74%) were diagnosed with febrile seizures, of which 45 (60.8%) were hospitalized. The majority of the patients 29 (64.4%), had simple febrile seizures, whereas 16 (35.6%) suffered from complex febrile seizures (Figure 1).

### Patient's clinical characteristics

The two groups of patients did not differ in age, gender, underlying disease, number of days of hospitalization, and poor sanity (all *p* > 0.05). However, we observed significant differences regarding previous history of FS, family history of FS, and maximum body temperature (all *p* < 0.05). Concerning COVID-19 associated clinical symptoms, cases did not differ from controls with regards to a stuffy nose, runny nose, headache, vomiting, diarrhea, non-consciousness, pulse rate, and SPO2 (all *p* > 0.05) but differed significantly in terms of cough, expectoration, dry rales, wet rales, and respiration rate (all *p* < 0.05). Detailed information is summarized in Table 1.

**Table 1:** Clinical characteristics of the patients.

Variables	Cases	Controls	*P*-value
No. (%)	45 (39.8)	68 (60.2)	
Age (months)	24.0 (15.5–41.0)	22.5 (3.0–68.3)	0.681
Range (months)	0–152	0–167	
Male/female	26 (57.8)/19 (42.2)	40 (58.8)/28 (41.2)	0.912
Underlying disease	20 (44.4)	19 (27.9)	0.071
Previous history of Fs	9 (20.0)	2 (2.9)	0.006
Family history of FS	5 (11.1)	0 (0.0)	0.009
Days of hospitalization	4.0 (4.0–5.5)	4.0 (3.0–5.8)	0.531
Maximum body temperature (°C)	39.5 ± 0.7	38.8 ± 1.1	<0.001
Poor sanity	8 (17.8)	14 (20.6)	0.712
Stuffy nose	2 (4.4)	8 (11.8)	0.311
Runny nose	5 (11.1)	13 (19.1)	0.255
Headache	0 (0.0)	3 (4.4)	0.275
Cough	10 (22.2)	49 (72.1)	<0.001
Expectoration	6 (13.3)	37 (54.4)	<0.001
Vomit	11 (24.4)	8 (11.8)	0.078
Diarrhea	0 (0.0)	4 (5.9)	0.150
Non-conscious	0 (0.0)	2 (2.9)	0.516
Dry rales	0 (0.0)	9 (13.2)	0.011
Wet rales	2 (4.4)	12 (17.6)	0.037
Respiration rate (breaths/minute)	27.2 ± 5.0	29.4 ± 5.9	0.043
Pulse rate (beats/minute)	118.3 ± 16.8	119.7 ± 17.7	0.280
SPO2 (%)	99.3 ± 1.2	92.4 ± 6.7	0.113

### Laboratory investigations

There were no statistically significant differences between the two groups in terms of C-reactive protein, interleukin 6, carbon dioxide, myoglobin, high-sensitivity troponin, and creatine kinase MB (all *p* > 0.05). Similar results were obtained for respiratory syncytial virus, influenza A and B, mycoplasma pneumonia, Epstein-Barr virus capsid antigen IgM, and cytomegalovirus IgM. In contrast, erythrocyte sedimentation rate, procalcitonin, and bacterial infection rates differed significantly (*p* < 0.05). Procalcitonin levels and bacterial infection rates were higher in cases than in controls, while ESR was lower in cases**.** Analysis of hematological parameters revealed that hemoglobin, platelets, lymphocytes, and eosinophils were significantly lower in cases than in controls (all *p* < 0.05). At the same time, neutrophil counts were significantly higher in cases than in controls (*p* < 0.05). However, there were no significant differences between the two groups regarding white blood cell counts, red blood cell counts, and monocyte counts (all *p* > 0.05). Liver, renal function tests and electrolyte levels showed no difference between cases and controls in terms of the levels of alanine transaminase, aspartate aminotransferase, total protein, albumin, globulin, creatinine, potassium, sodium, chloride, and calcium levels (all *p* > 0.05), while, blood urea nitrogen levels were significantly higher in cases than control (all *p* < 0.05). Detailed information is presented in Table 2.

**Table 2:** Comprehensive laboratory investigations.

Variables	Cases (*n* = 45)	Controls (*n* = 68)	*P*-value
CRP (mg/L)	3.63 (1.38–11.38)	3.25 (0.79–13.5)	0.824
ESR on admission (mm/h)	3.50 (2.00–6.00)	7.50 (4.50–13.75)	<0.001
PCT (ng/ml)	0.29 (0.09–0.96)	0.10 (0.05–0.26)	0.002
IL6 (pg/ml)	18.2 (11.0–27.5)	10.1 (5.2–26.2)	0.151
CO2	20.0 (17.8–22.0)	19.4 (17.7–21.9)	0.490
Myoglobin (ng/ml)	33.1 (24.0–58.7)	26.7 (16.6–40.1)	0.064
High-sensitivity troponin	3.80 (1.45–13.40)	4.05 (0.90–15.9)	0.554
CKMB (ng/L)	1.30 (0.98–3.50)	1.40 (0.80–3.70)	0.716
RSV	0 (0.0)	1 (0.9)	1.000
IFA	10 (23.3)	9 (13.8)	0.209
IFB	18 (41.9)	18 (27.7)	0.126
MP	10 (23.3)	13 (20.0)	0.686
EBV capsid antigen IgM	0.49 (0.36–0.85)	0.50 (0.25–0.79)	0.531
>3.0	0 (0.0)	2 (4.5)	0.498
CMV-IgM	0.171 (0.157–0.186)	0.186 (0.148–0.258)	0.377
>1.0	0 (0.0)	2 (4.5)	0.498
Bacterial infection	15 (33.3)	11 (16.2)	0.034
WBC (10^9/L^)	6.07 (4.76–8.20)	6.36 (4.50–9.50)	0.369
RBC (10^12/L^)	4.30 (3.93–4.46)	4.40 (4.08–4.81)	0.192
Hb (g/dl)	117 (109–122)	122 (112–129)	0.025
PLT (10^9/L^)	206 (178–228)	244 (195–295)	0.004
N (%)	72.7 (53.0–82.8)	45.5 (31.6–61.2)	<0.001
L (%)	15.1 (7.8–31.6)	33.5 (17.9–51.3)	<0.001
M (%)	10.1 (6.9–13.9)	12.5 (8.1–18.4)	0.056
E (%)	0.10 (0.00–0.40)	0.80 (0.20–3.20)	<0.001
ALT (U/L)	19.0 (15.0–23.0)	22.0 (14.0–35.0)	0.122
AST (U/L)	43.0 (35.3–56.0)	48.0 (34.0–67.0)	0.380
TP (g/L)	63.5 (59.5–68.8)	64.7 (58.2–70.0)	0.649
ALB (g/L)	42.1 (40.0–45.3)	41.3 (37.9–44.5)	0.081
GLB (g/L)	21.3 (19.9–23.9)	23.3 (19.5–27.7)	0.198
BUN (mmol/L)	4.60 (3.52–5.43)	3.40 (2.85–4.75)	<0.001
Cr (umol/L)	29.6 (25.7–35.5)	32.5 (25.9–38.5)	0.305
Glucose (mg/dl)	5.67 (4.85–7.06)	5.35 (4.62–6.69)	0.222
K (mmol/L)	4.41 (3.96–5.05)	4.79 (4.18–5.36)	0.052
Na (mmol/L)	135 (132–137)	136 (134–138)	0.072
Cl (mmol/L)	104 (101–105)	104 (102–106)	0.231
Ca2 (mmol/L)	2.38 (2.28–2.53)	2.43 (2.27–2.49)	0.889

CRP, C-reactive protein; ESR, erythrocyte sedimentation rate; PCT, procalcitonin; IL6, interleukin 6; CO2, carbon dioxide; CKMB, creatine kinase MB; RSV, respiratory syncytial virus; IFA, influenza A; IFB, influenza B; MP, mycoplasma pneumonia; EBV, Epstein-Barr virus; CMV-IgM, cytomegalovirus immunoglobulin M; bacterial infection; WBC, white blood cells; RBC, red blood cells; Hb, hemoglobin; PLT, platelets; N, neutrophil; L, lymphocytes; M, monocyte; E, eosinophils; ALT, alanine transaminase; AST, aspartate aminotransferase; TP, total protein; ALB, albumin; GLB, globulin; BUN, blood urea nitrogen; Cr, creatinine; K, potassium; Na, sodium; Cl, chloride; Ca2, calcium.

### Medication use

There was no difference in the prescription of piperacillin, tazobactam, azithromycin, clindamycin, ceftriaxone, cefoperazone, sulbactam, ribavirin, and ganciclovir between cases and controls (all *p* > 0.05). In contrast, more children in the case group were prescribed antiflu medications than those in the control group (*p* < 0.05). Additionally, we did not observe any significant differences between case and control with regards to intravenous corticosteroids, such as hydrocortisone, dexamethasone, or nebulizers, including beclomethasone dipropionate inhalation, ipratropium bromide inhalation, albuterol inhalation, acetylcysteine inhalation or expectorants such as ambroxol oral medicine, oral montelukast sodium, non-invasive breathing, oxygen with a mask or nasal cannula, invasive breathing (all *p* > 0.05). In contrast, fewer children in the case group were given methylprednisolone, sodium succinate injection, budesonide inhalation, and ambroxol injection than those in the control group (*p* < 0.05). Detailed information is presented in Table 3.

**Table 3:** Medications used.

Variables	Cases (*n* = 45)	Controls (*n* = 68)	*P*-value
Use of antibiotics	27 (60.0)	35 (51.5)	0.372
Piperacillin tazobactam	21 (46.7)	23 (33.8)	0.170
Azithromycin	6 (13.3)	12 (17.6)	0.540
Clindamycin	0 (0.0)	5 (7.4)	0.155
Ceftriaxone	1 (2.2)	0 (0.0)	0.398
Cefoperazone sulbactam	2 (4.4)	1 (1.5)	0.562
Oral cephalosporin	0 (0.0)	2 (2.9)	0.516
Use of antivirals	18 (40.0)	14 (20.6)	0.025
Anti-flu medicine	18 (40.0)	11 (16.2)	0.005
Ribavirin	0 (0.0)	2 (2.9)	0.516
Ganciclovir	0 (0.0)	2 (2.9)	0.516
Intravenous corticosteroids	5 (11.1)	19 (27.9)	0.032
Methylprednisolone sodium succinate needle	0 (0.0)	17 (25.0)	<0.001
Hydrocortisone needles	0 (0.0)	1 (1.5)	1.000
Dexamethasone needles	5 (11.1)	2 (2.9)	0.113
Nebulizer	21 (46.7)	50 (73.5)	0.004
budesonide inhalation	20 (44.4)	50 (73.5)	0.002
beclomethasone dipropionate inhalation	1 (2.2)	1 (1.5)	1.000
Ipratropium bromide inhalation	3 (6.7)	13 (19.1)	0.063
Albuterol inhalation	1 (2.2)	1 (1.5)	1.000
Acetylcysteine inhalation	0 (0.0)	5 (7.4)	0.155
Expectorants	10 (22.2)	33 (48.5)	0.005
Ambroterol oral medicine	8 (17.8)	13 (19.1)	0.858
Ambroxol injection	2 (4.4)	25 (36.8)	<0.001
Oral montelukast sodium	2 (4.4)	4 (5.9)	1.000
Non-invasive breathing	7 (15.6)	11 (16.2)	0.930
Oxygen with mask or nasal cannula	7 (15.6)	11 (16.2)	0.930
Invasive breathing	0 (0.0)	0 (0.0)	–

### Predictors of febrile seizures in COVID-19 infected children

Lastly, we conducted a Poisson regression analysis to determine factors that can predict the development of febrile seizures in children infected with COVID-19. We adjusted for cough, maximum body temperature, history of previous seizures, eosinophil count, hemoglobin levels, procalcitonin levels, neutrophil count, platelet count, and blood urea nitrogen.

In a minimally adjusted model (model 1), children with COVID-19 and cough were less likely to experience FS than their controls, adjusted [RR 0.309; (95% CI: 0.170–0.564), *p *< 0.001], while those with high body temperatures and previous history of febrile seizures were more likely to experience febrile seizures than their controls, adjusted [RR 1.474; (95% CI: 1.196–1.818), *p *< 0.001], and [RR 1.421; (95% CI: 1.088–1.855), *p *= 0.010], respectively.

Using the moderately adjusted model (model 2), which was adjusted for eosinophil count, hemoglobin level, neutrophil count, platelet count, and blood urea nitrogen, children with low eosinophil counts and hemoglobin levels had a lower risk of developing FS [RR 0.494; (95% CI: 0.311–0.783), *p *= 0.003] and [RR 0.979 (95% CI: 0.959–0.999), *p *= 0.044], respectively. In comparison, those with higher procalcitonin levels and neutrophil counts had a higher risk of experiencing FS than those without [RR 1.140; (95% CI: 1.043–1.246), *p *= 0.048], and [RR 1.015; (95% CI: 1.000–1.029), *p *= 0.048], respectively.

After adjusting for all these factors in (model 3), children with COVID-19 with low eosinophil counts [RR 0.428 (95% CI: 0.275–0.666), *p *< 0.001], low hemoglobin levels [RR 0.978 (95% CI: 0.960–0.996), *p *= 0.015], and cough [RR 0.473 (95% CI: 0.252–0.890), *p *= 0.020], had a lower risk of developing FS. Meanwhile, children with COVID-19 with higher procalcitonin levels [RR 1.140 (95% CI: 1.043–1.246), *p *= 0.004] and neutrophil counts [RR 1.017 (95% CI: 1.003–1.031), *p *= 0.015] had a higher risk of experiencing FS than those without (Table 4).

**Table 4:** Adjusted poisson regression models for predicting febrile seizures.

Models	Adjusted RR (95% CI)	Wald chi-square	*P* value
Model 1			
Cough	0.309 (0.170–0.564)	14.658	<0.001
Maximum body temperature (1°C increase)	1.474 (1.196–1.818)	13.203	<0.001
History of previous seizures	1.421 (1.088–1.855)	6.672	0.010
Model 2			
E%	0.494 (0.311–0.783)	8.988	0.003
Hb	0.979 (0.959–0.999)	4.046	0.044
PCT	1.137 (1.001–1.292)	3.912	0.048
N%	1.015 (1.000–1.029)	3.897	0.048
PLT	0.996 (0.991–1.001)	2.639	0.104
BUN	1.085 (0.867–1.359)	0.508	0.476
Model 3			
E%	0.428 (0.275–0.666)	14.109	<0.001
PCT	1.140 (1.043–1.246)	8.304	0.004
N%	1.017 (1.003–1.031)	5.895	0.015
Hb	0.978 (0.960–0.996)	5.888	0.015
Cough	0.473 (0.252–0.890)	5.387	0.020
History of previous seizures	1.290 (0.837–1.990)	1.328	0.249
Maximum body temperature (1°C increase)	0.817 (0.059–1.193)	1.099	0.295

E, eosinophil count; Hb, hemoglobin level; N, neutrophil count; PLT, platelet count; and BUN, blood urea nitrogen.

## Discussion

In this case-control study, we examined the incidence of febrile seizure among SARS-CoV-2-Omicron-infected children, their admission rate, risk factors associated with febrile seizures among the infected children, and their treatment modalities. Our findings revealed that 5.74% of the children had febrile seizures, and approximately 60.8% were hospitalized. These findings suggest that both COVID-19 associated OPD visits and the incidence of febrile seizures among the COVID-19-infected children rapidly increased during the Omicron period. Our results are consistent with those of Cloete et al., who reported a rapid rise in pediatric COVID-19 hospitalizations in the early stages of the Omicron wave in South Africa (25). Furthermore, a Korean study by Joung et al. demonstrated that 7.0% of COVID-19 infected Korean children developed COVID-19-associated febrile seizures during the Omicron period (26).

In August 2021, during the highly transmissible “delta variant” wave, the Chinese government adopted the “Dynamic zero-COVID” policy to stem the spread of the disease (27). During that period, pediatric seizure-related hospitalizations dropped drastically. For instance, a study in Hong Kong demonstrated a significant reduction in seizure-related emergency department visits in 2020 [RR 0.39 (95% CI: 0.174–0.526), *p* = 0.001]. Furthermore, the rate of febrile seizure-related admissions for children aged 0–6 decreased by more than 20% from the previous year (28). However, on December 7, 2022, China abruptly abandoned its zero-COVID policy, leading to a surge in COVID-19 outbreaks nationwide, with the mutated Omicron variant as the predominant strain (1). Since then, there has been a significant increase in pediatric infections with the SARS-CoV-2 Omicron variant and subsequently increased incidence of febrile seizures (2).

This study had no statistical difference in the ages of cases and controls (*p *> 0.05). However, children with febrile seizures had a slightly higher median (IQR) age than the controls, 24.0 (15.5–41.0) vs. 22.5 (3.0–68.3) months, respectively. Our findings are supported by Juong et al., who also found that children with COVID-19 and febrile seizures were generally older than those without febrile seizures; median (IQR) age 33.0 (22.0–60.0) and 23.0 (14.5–31.5) months, although their control group was COVID-19 negative children (26). In addition, evidence suggests that sex can influence the differentiation and maintenance of virus-driven *T* cells and antiviral immune response in tissues (29). This study had no statistical difference between the groups regarding sex distribution. Similarly, the underlying disease, number of days of hospitalization, and poor sanity of both groups did not statistically differ.

Published literature alludes to genetic predispositions for FS. For instance, Veisani et al.'s meta-analysis concluded that family history is strongly associated with febrile seizure (30). In this study, we observed a previous history of FS and a family history of FS in 20.0% and 11.1% of the cases and only 2.9% and 0% of the controls, respectively. Based on these findings, genetic factors and shared environmental exposures may play an essential role in the development of FS.

According to Taytard and colleagues, children infected with the Omicron variant have significantly higher body temperatures than those infected with the Delta variant (30). The higher peak body temperature caused by SARS-CoV-2 infection during the Omicron period may have contributed to increased febrile seizures. Despite both cases and controls in our study being positive for the Omicron variant, the cases had significantly higher body temperatures than the controls; mean (±SD) body temperature, 39.5 ± 0.7°C and 38.8 ± 1.1°C, respectively. These findings are consistent with those of Margaretha et al. (31), who found mean temperatures of 39.01 ± 0.56°C and 38.64 ± 0.45°C for children with and without FS, respectively (31). Meanwhile, Leung et al. observed that FS tended to occur when body temperatures exceeded 39°C (32).

Other clinical symptoms associated with COVID-19 were not significantly different between cases and controls, except for cough, expectoration, dry rales, wet rales, and respiration rate, which were significantly higher in controls. In most cases, COVID-19 affects children in a mild form. Fever, dry cough, and fatigue are some of the common symptoms experienced by children (33). In previous studies, the cough has been associated with high intrathoracic pressure, which can result in cough-induced syncope and fainting (34). However, our study found that cough was associated with a lower risk of febrile seizures [RR 0.473 (95% CI: 0.252–0.890), *p *= 0.020]. Children with COVID-19 and cough were less likely to experience febrile seizures than controls, whereas those who had a high fever were more likely to experience febrile seizures. This suggests that fever is more closely associated with FS than respiratory symptoms.

Regarding the laboratory results, bacterial infection rates were higher in cases than in controls. Bacterial infection was observed in 33.3% of children with FS and 16.2% of controls. Our findings are consistent with Jarrett et al. (35), who found a higher prevalence of bacterial infection in cases than thin e control. Similarly, procalcitonin levels (PCT) were higher in cases than in controls, with median PCT levels of 0.29 ng/ml in cases ag/ml in controls. A similar finding was found by Murakami et al. (36) who found increased levels of PCT in convulsion patients. Increased levels of procalcitonin were first observed in 1993 in patients with sepsis, and today it is a widely used biomarker for serious bacterial infections (37). PCT is a natural prohormone of calcitonin synthesized by C-cells of the thyroid glands (38). Its production increases in patients with septicemia and infections caused by gram-negative bacteria due to inflammatory cytokines, such as tumor necrosis factor-alpha and interleukin-6 (IL-6) (39). This study observed gram-negative mycoplasma pneumonia co-infections in 23.3% of cases compared to 20% of controls. Furthermore, the erythrocyte sedimentation rate was significantly lower in cases than in controls, which corresponds with the findings of Abbasi et al. (40).

According to a study by Pisacane et al., children with FS had significantly higher rates of iron deficiency anaemia than their non-FS counterparts (41). Interestingly, Talebian et al. revealed that anaemic children had a reduced incidence of febrile seizure compared to children without anaemia (42). In support of this finding, Yousefichaijan and colleagues found that iron deficiency prevents febrile convulsions in children and likely increases the threshold of neuronal excitation during fevers (43). In line with these studies, our findings revealed that cases had lower haemoglobin levels than controls and had a lower risk of developing FS [RR, (0.979; 95% CI: 0.959–0.999), *p* = 0.044]. Similarly, children with low eosinophil counts had a lower risk of developing FS [RR 0.494; (95% CI: 0.311–0.783), *p *= 0.003] than the controls. Eosinophils play an essential role in allergic inflammation, but no evidence has been found linking them to FS (44).

Moreover, in a recent study by Romanowska et al., the platelet and lymphocyte count of children with FS were significantly lower than those of controls. In contrast, the neutrophil counts were significantly higher (45). In line with the study, our study found that the platelet and lymphocyte counts were significantly lower in cases than in controls. Meanwhile, neutrophil counts were significantly higher in cases than in controls. It has been determined that neutrophil-lymphocyte ratio and mean platelet volume are novel inflammatory biomarkers associated with the development of FS in children (44).

Regarding liver and renal function tests and serum electrolytes, there was no significant difference between cases and controls, other than blood urea nitrogen level, which was significantly higher in cases than in controls. This finding is supported by Abbasi et al., who also found that patients with febrile seizures had substantially higher levels of BUN and creatinine than those without febrile seizures (40). It has been shown that patients with a history of illnesses, such as vomiting and diarrhea or signs of dehydration, may affect their renal function tests (46). In this study, 24.4% of the cases and 11.8% of the controls reported vomiting during admission. While it has been shown that hyponatremia is associated with febrile seizures (47), another study by Yousefichaija et al. has found that children with febrile seizures have normal electrolyte levels. And therefore, it is not necessary to routinely test the electrolyte levels of all patients with simple febrile seizures (48). This study found no differences between cases and controls in electrolyte levels.

The prescription of antibiotics and antivirals was not significantly different between the two groups, except for the antiflu medications, where cases got more than the controls. In contrast, fewer children in the case group were given corticosteroids and expectorants than controls. The increasing use of antiflu medications could be attributed to SARS-CoV-2 co-infection with influenza A and B viruses. A meta-analysis by Masoud Dadashi et al. showed that influenza infection was 0.8% in patients with confirmed COVID-19, while the frequency of influenza virus co-infection among patients with COVID-19 was 4.5% in Asia and 0.4% in North America (49). In line with these findings, we observed a higher incidence of influenza A; 23.3% vs. 13.8%, and influenza B; 41.9% vs. 27.7% among cases than controls, respectively.

Strengths and limitations: This study had a few distinct advantages worth highlighting: (1) It was the first study to examine the incidence of febrile seizures in children infected with the SARS-CoV-2-Omicron variant in Hubei, China. (2) It further examined detailed clinical presentations and risk factors associated with febrile seizures in children infected with the SARS-CoV-2-Omicron variant. However, the following limitations existed: (1) Sample size was rather small and this could limit the study's power. (2) The study was retrospectively conducted thus it carried the inevitable disadvantages of retrospective studies such as recall bias and missing information. (3) The study was a single-center study, so the findings cannot be generalized to make inferences on the incidence of FS among children infected with COVID-19 across the country.

## Conclusion

In summary, a significant increase in febrile seizures was observed in children with SARS-CoV-2-Omicron infection. Approximately 5.74% of participants with COVID-19 suffered febrile seizures. A higher body temperature, a history of FS, a higher procalcitonin level and a higher neutrophil count were all associated with an increased risk of FS in children with COVID-19. The risk of developing FS was lower in children with COVID-19 who had low eosinophil counts, and low hemoglobin levels. More children in the case were prescribed antiflu medications than those in the control group. In contrast, fewer children in the case group were given corticosteroids and expectorants than those in the control group.

## Data Availability

The original contributions presented in the study are included in the article/Supplementary Material, further inquiries can be directed to the corresponding authors.

## References

[1] Zheng L Liu S Lu F. Impact of national omicron outbreak at the end of 2022 on the future outlook of COVID-19 in China. Emerg Microbes Infect. (2023) 12(1):2191738. 10.1080/22221751.2023.2191738 36920784 PMC10044155

[2] Shen N Wu YF Chen YW Fang XY Zhou M Wang WY Clinical characteristics of pediatric cases infected with the SARS-CoV-2 Omicron variant in a tertiary children’s medical centre in Shanghai, China. World J Pediatr. (2023) 19(1):87–95. 10.1007/s12519-022-00621-6 36251118 PMC9574794

[3] Zhou JG Lu YM Wang LB Yu H Zhang T Chen YW Pediatric patients in the new wave of SARS-CoV-2 infection in Shanghai, China. World J Pediatr. (2022) 18(9):579–81. 10.1007/s12519-022-00570-0 35713812 PMC9204678

[4] Li Y-C Ma Z Zhong H-Y You H-L. Clinical characteristics of children with Omicron SARS-CoV-2 infection in Changchun, China from March to April 2022: a retrospective study. Front Pediatr. (2022) 10:990944. 10.3389/fped.2022.990944 36458144 PMC9705729

[5] Bahl A Mielke N Johnson S Desai A Qu L. Severe COVID-19 outcomes in pediatrics: an observational cohort analysis comparing alpha, Delta, and Omicron variants. Lancet Reg Health Am. (2023) 18:100405. 10.1016/j.lana.2022.100405 36474521 PMC9714340

[6] Cloete J Kruger A Masha M du Plessis NM Mawela D Tshukudu M Paediatric hospitalisations due to COVID-19 during the first SARS-CoV-2 Omicron (B.1.1.529) variant wave in South Africa: a multicentre observational study. Lancet Child Adolesc Health. (2022) 6(5):294–302. 10.1016/S2352-4642(22)00027-X 35189083 PMC8856663

[7] Khemiri H Ayouni K Triki H Haddad-Boubaker S. SARS-CoV-2 infection in pediatric population before and during the Delta (B.1.617.2) and Omicron (B.1.1.529) variants era. Virol J. (2022) 19(1):144. 10.1186/s12985-022-01873-4 36076271 PMC9452867

[8] Standard B. Covid cases in children rose in Omicron wave in Europe, shows data. (2022). Available at: https://www.business-standard.com/article/international/covid-cases-in-children-rose-in-omicron-wave-in-europe-shows-data-122031701297_1.html (Cited Apr 17, 2023).

[9] Acker KP Levine DA Varghese M Nash KA RoyChoudhury A Abramson EL Indications for hospitalization in children with SARS-CoV-2 infection during the Omicron wave in New York City. Children. (2022) 9(7):1043. 10.3390/children9071043 35884027 PMC9320728

[10] Brewster RC Parsons C Laird-Gion J Hilker S Irwin M Sommerschield A COVID-19-associated croup in children. Pediatrics. (2022) 149(6):e2022056492. 10.1542/peds.2022-056492 35257175

[11] Choi YY Kim YS Lee SY Sim J Choe YJ Han MS. Croup as a manifestation of SARS-CoV-2 Omicron variant infection in young children. J Korean Med Sci. (2022) 37(20):e140. 10.3346/jkms.2022.37.e140 35607737 PMC9127433

[12] Sharma S Agha B Delgado C Walson K Woods C Gonzalez MD Croup associated with SARS-CoV-2: pediatric laryngotracheitis during the Omicron surge. J Pediatr Infect Dis Soc. (2022) 11(8):371–4. 10.1093/jpids/piac032 PMC9426860 35512450

[13] Tunҫ EM Koid Jia Shin C Usoro E Thomas-Smith SE Trehan I Migita RT Croup during the coronavirus disease 2019 Omicron variant surge. J Pediatr. (2022) 247:147–9. 10.1016/j.jpeds.2022.05.006 35551925 PMC9085454

[14] Han MJ Heo JH Hwang JS Jang YT Lee M Kim SJ. Incidence of febrile seizures in children with COVID-19. J Clin Med. (2023) 12(3):1076. 10.3390/jcm12031076 36769723 PMC9918282

[15] Iijima H Kubota M Ogimi C. Change in seizure incidence in febrile children with COVID-19 in the era of Omicron variant of concern. J Pediatr Infect Dis Soc. (2022) 11(11):514–7. 10.1093/jpids/piac085 35984115

[16] Cadet K Boegner J Ceneviva GD Thomas NJ Krawiec C. Evaluation of febrile seizure diagnoses associated with COVID-19. J Child Neurol. (2022) 37(5):410–5. 10.1177/08830738221086863 35286175 PMC9086105

[17] Leung AK Hon KL Leung TN. Febrile seizures: an overview. Drugs Context. (2018) 7:212536. 10.7573/dic.212536 30038660 PMC6052913

[18] Delpisheh A Veisani Y Sayehmiri K Fayyazi A. Febrile seizures: etiology, prevalence, and geographical variation. Iran J Child Neurol. (2014) 8(3):30–7. 25143771 PMC4135278

[19] Kaputu Kalala Malu C Mafuta Musalu E Dubru JM Leroy P Tomat AM Misson JP. Epidemiology and characteristics of febrile seizures in children. Rev Med Liege. (2013) 68(4):180–5. 23755708

[20] Berg AT Shinnar S Shapiro ED Salomon ME Crain EF Hauser WA. Risk factors for a first febrile seizure: a matched case-control study. Epilepsia. (1995) 36(4):334–41. 10.1111/j.1528-1157.1995.tb01006.x 7541745

[21] Chung B Wong V. Relationship between five common viruses and febrile seizure in children. Arch Dis Child. (2007) 92(7):589–93. 10.1136/adc.2006.110221 17284480 PMC2083759

[22] Carman KB Calik M Karal Y Isikay S Kocak O Ozcelik A Viral etiological causes of febrile seizures for respiratory pathogens (EFES study). Hum Vaccines Immunother. (2019) 15(2):496–502. 10.1080/21645515.2018.1526588 PMC6422444 30235060

[23] Kukuruzovic M. 391 Febrile convulsions and influenza A or B- are there differences? Arch Dis Child. (2021) 106(Suppl 2):A164. 10.1136/archdischild-2021-europaediatrics.391

[24] Subcommittee on Febrile Seizures, American Academy of Pediatrics. Neurodiagnostic evaluation of the child with a simple febrile seizure. Pediatrics. (2011) 127(2):389–94. 10.1542/peds.2010-3318 21285335

[25] Cloete J Kruger A Masha M du Plessis NM Mawela D Tshukudu M Rapid rise in paediatric COVID-19 hospitalisations during the early stages of the omicron wave, Tshwane District, South Africa. medRxiv (2021). s2352-4642 (22)00027-x:2021.12.21.21268108. 10.1101/2021.12.21.21268108 Available at: https://www.medrxiv.org/content/10.1101/2021.12.21.21268108v1 (Cited April 26, 2023).

[26] Joung J Yang H Choi YJ Lee J Ko Y. The impact of Omicron wave on pediatric febrile seizure. J Korean Med Sci. (2022) 38(3):e18. 10.3346/jkms.2023.38.e18 PMC9842486 36647218

[27] Liu J Liu M Liang W. The dynamic COVID-zero strategy in China. China CDC Wkly. (2022) 4(4):74–5. 10.46234/ccdcw2022.015 35186372 PMC8837441

[28] Chiu TGA Leung WCY Zhang Q Lau EHY Ho RWH Chan HSS Changes in pediatric seizure-related emergency department attendances during COVID-19—a territory-wide observational study. J Formos Med Assoc. (2021) 120(8):1647–51. 10.1016/j.jfma.2020.11.006 33248859 PMC7680012

[29] Poon MML Byington E Meng W Kubota M Matsumoto R Grifoni A Heterogeneity of human anti-viral immunity shaped by virus, tissue, age, and sex. Cell Rep. (2021) 37(9):110071. 10.1016/j.celrep.2021.110071 34852222 PMC8719595

[30] Taytard J Prevost B Schnuriger A Aubertin G Berdah L Bitton L SARS-CoV-2 B.1.1.529 (Omicron) variant causes an unprecedented surge in children hospitalizations and distinct clinical presentation compared to the SARS-CoV-2 B.1.617.2 (Delta) variant. Front Pediatr. (2022) 10:932170. 10.3389/fped.2022.932170 35832582 PMC9271577

[31] Margaretha L Masloman N. Correlation between serum zinc level and simple febrile seizure in children. Paediatr Indones. (2010) 50(6):326–30. 10.14238/pi50.6.2010.326-30

[32] Leung AK Robson WL. Febrile convulsions. How dangerous are they? Postgrad Med. (1991) 89(5):217–8. 221–2, 224. 10.1080/00325481.1991.11700905 2008400

[33] Ludvigsson JF. Systematic review of COVID-19 in children shows milder cases and a better prognosis than adults. Acta Paediatr. 2020 109(6):1088–95. 10.1111/apa.15270 32202343 PMC7228328

[34] Waldmann V Combes N Narayanan K Sharifzadehgan A Bouzeman A Beganton F Cough syncope. Am J Med. (2017) 130(7):e295–6. 10.1016/j.amjmed.2017.01.050 28238688

[35] Jarrett OO Fatunde OJ Osinusi K Lagunju IA. Prevalence of bacteraemia amongst children with febrile seizures at the University College Hospital, Ibadan. Ann Ib Postgrad Med. 2018 16(2):170–3. 31217776 PMC6580404

[36] Murakami H Naraba H Gondo T Mochizuki M Nakano H Takahashi Y Diagnostic value of serum procalcitonin in patients with convulsion in emergency department, an observational study. Antibiotics. (2020) 9(10):683. 10.3390/antibiotics9100683 33050085 PMC7599961

[37] Assicot M Gendrel D Carsin H Raymond J Guilbaud J Bohuon C. High serum procalcitonin concentrations in patients with sepsis and infection. Lancet Lond Engl. (1993) 341(8844):515–8. 10.1016/0140-6736(93)90277-N PMC7141580 8094770

[38] Maruna P Nedelníková K Gürlich R. Physiology and genetics of procalcitonin. Physiol Res. (2000) 49(Suppl 1):S57–61. 10984072

[39] Dandona P Nix D Wilson MF Aljada A Love J Assicot M Procalcitonin increase after endotoxin injection in normal subjects. J Clin Endocrinol Metab. (1994) 79(6):1605–8. 10.1210/jcem.79.6.7989463 7989463

[40] Abbasi E Ghazavi A Firoozi A. Comparison of laboratory parameters in children admitted with febrile seizures and fever without localized sign in Urmia Motahari Hospital, Iran, 2015 until 2020. Med J Tabriz Univ Med Sci. (2022) 44(1):47–54. 10.34172/mj.2022.013

[41] Pisacane A Sansone R Impagliazzo N Coppola A Rolando P D'Apuzzo A Iron deficiency anaemia and febrile convulsions: case-control study in children under 2 years. BMJ. (1996) 313(7053):343. 10.1136/bmj.313.7053.343 8760744 PMC2351736

[42] Talebian A Momtazmanesh N. Febrile seizure and anemia. Iran J Child Neurol. (2007) 2(1):31–3.

[43] Yousefichaijan P Eghbali A Rafeie M Sharafkhah M Zolfi M Firouzifar M. The relationship between iron deficiency anemia and simple febrile convulsion in children. J Pediatr Neurosci. (2014) 9(2):110–4. 10.4103/1817-1745.139276 25250062 PMC4166829

[44] Liu Z Li X Zhang M Huang X Bai J Pan Z The role of mean platelet volume/platelet count ratio and neutrophil to lymphocyte ratio on the risk of febrile seizure. Sci Rep. (2018) 8:15123. 10.1038/s41598-018-33373-3 30310107 PMC6181908

[45] Gontko-Romanowska K Żaba Z Panieński P Steinborn B Szemień M Łukasik-Głębocka M The assessment of laboratory parameters in children with fever and febrile seizures. Brain Behav. (2017) 7(7):e00720. 10.1002/brb3.720 28729928 PMC5516596

[46] Taylor K Jones EB. Adult dehydration. StatPearls. Treasure Island, FL: StatPearls Publishing (2023). Available at: http://www.ncbi.nlm.nih.gov/books/NBK555956/ (Cited April 29, 2023). 32310416

[47] Miyagi Y Sasano T Kato H Kin K. Hyponatremia and recurrent febrile seizures during febrile episodes: a meta-analysis. Cureus. (2022) 14(4):e24398. 10.7759/cureus.24398 35619851 PMC9126426

[48] Yousefichaijan P Dorreh F Abbasian L Pakniyat AG. Assessing the prevalence distribution of abnormal laboratory tests in patients with simple febrile seizure. J Pediatr Neurosci. (2015) 10(2):93–7. 10.4103/1817-1745.159180 26167207 PMC4489076

[49] Dadashi M Khaleghnejad S Abedi Elkhichi P Goudarzi M Goudarzi H Taghavi A COVID-19 and influenza co-infection: a systematic review and meta-analysis. Front Med. (2021) 8:681469. 10.3389/fmed.2021.681469 PMC8267808 34249971

